# Premature immunosenescence in AYA gastrointestinal cancer: The metabolic-inflammatory-hormonal axis as an integrative framework: A systematic review

**DOI:** 10.3892/mi.2026.338

**Published:** 2026-09-01

**Authors:** Jie Wen, Haiyan Yu

**Affiliations:** Department of Gastroenterology, Peking University First Hospital Taiyuan Branch (Taiyuan Central Hospital), Taiyuan, Shanxi 030032, P.R. China

**Keywords:** adolescents and young adults, gastrointestinal cancer, premature immunosenescence, tumor microenvironment, metabolic-inflammatory-hormonal axis, precision oncology, tumor mutational burden, gut microbiome

## Abstract

The global rise in gastrointestinal (GI) cancer among adolescents and young adults (AYAs; aged 15-39) represents a striking departure from traditional age-dependent epidemiology. In the present systematic review, it is proposed that premature immunosenescence may be a contributing driver of this trend and introduces the metabolic-inflammatory-hormonal axis as a working framework to explore these observations. Findings from PubMed/MEDLINE (1990-2024) were synthesized, focusing on high-impact studies and systematic reviews from the last 5 years, using keywords related to young-onset GI cancer, immunosenescence and metabolic inflammation. A PRISMA screening process was employed, with defined inclusion/exclusion criteria. The present systematic review identified several interconnected mechanisms that may drive premature immune aging in AYAs: i) T-cell exhaustion and thymic involution; ii) chronic inflammation fueled by the senescence-associated secretory phenotype and tumor-derived vesicles; and iii) metabolic perturbations, such as cholesterol buildup and lactate-mediated immunosuppression. Collectively, these changes may create an immunosuppressive microenvironment enriched with regulatory T cells, myeloid-derived suppressor cells and M2 macrophages. The metabolic-inflammatory-hormonal (M-I-H) axis integrates these pathways, potentially driving immune dysfunction independent of chronological age. The present systematic review emphasizes that this framework remains largely hypothetical, with numerous proposed associations awaiting direct experimental validation in AYA populations. Biomarkers including polymerase ε/polymerase δ1 mutations, microsatellite instabilityhigh/deficient mismatch repair-status, tumor mutational burden, circulating tumor DNA, and immune gene signatures may help guide precision therapy, although their specific relationship to premature immunosenescence requires further investigation. It was proposed in the present systematic review that targeting this axis, through metabolic modulation, anti-inflammatory strategies, hormonal interventions, or microbiota engineering, holds promise for treating AYA GI cancer. Ultimately, validating plasma biomarkers for early detection and assessing these combinatorial approaches in prospective trials will be critical to addressing this growing clinical challenge.

## Introduction

Gastrointestinal (GI) malignancies have traditionally been associated with aging. For decades, clinicians observed incidence curves that rose exponentially after the age of 60 years, with few cases observed in younger populations. However, something appears to have shifted. Over the past two decades, approximately since the early 2000s, clinicians in both Western and Asian centers began noticing an unsettling pattern: An increase in the number of adolescent and young adult (AYA) patients, often in their 20s and 30s, presenting with advanced colorectal or gastric cancer.

**Figure 1:**
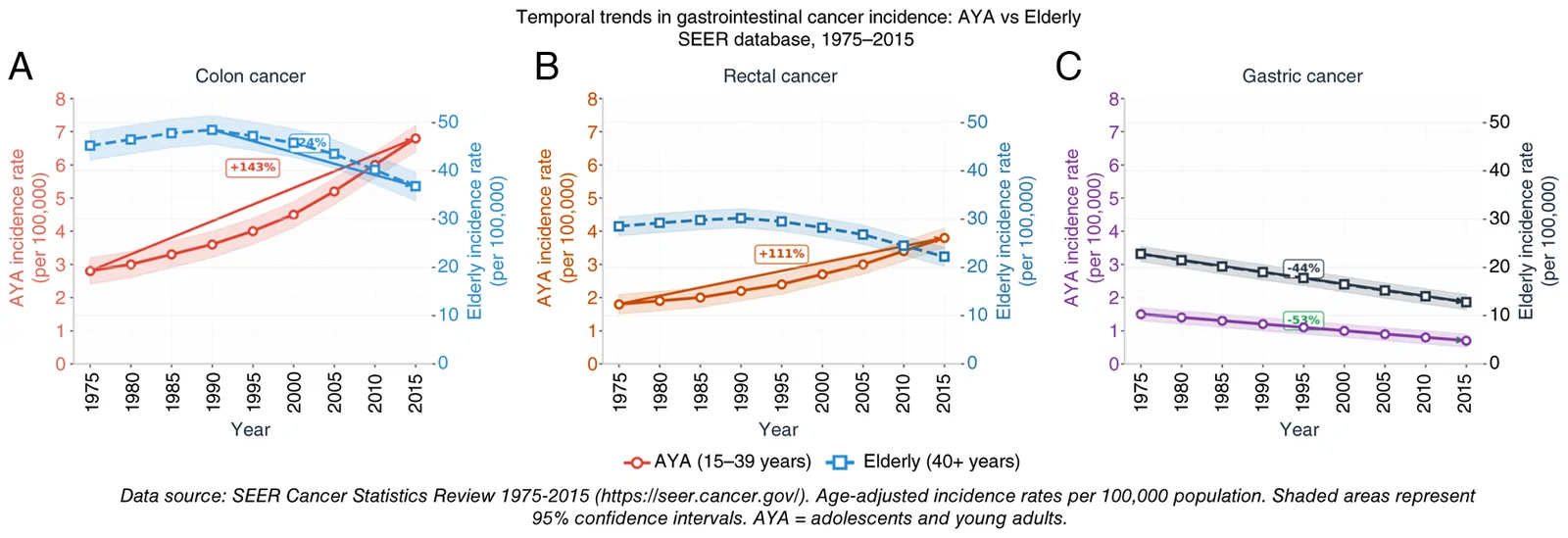
Divergent trends in gastrointestinal cancer incidence between AYA (15-39 years) and elderly (≥40 years) populations, SEER 1975-2015. (A) Colon and (B) rectal cancers showed marked increases in AYA incidence (+143 and +111%, respectively) vs. declines in elderly populations, with clear divergence after 1995. (C) Gastric cancer decreased in both groups. Shaded areas, 95% confidence intervals. This divergence supports the need to consider age-specific biological mechanisms, including premature immunosenescence, rather than attributing the trend solely to enhanced detection. The birth-cohort pattern visible in panels A and B is consistent with a generational shift in environmental or biological risk factors converging on the M-I-H axis. AYA, adolescents and young adults; SEER, Surveillance, Epidemiology, and End Results program; M-I-H, metabolic-inflammatory-hormonal.

The numbers support this. Individuals born after 1960 demonstrate a >60% increase in the risk of AYA-onset colorectal and gastric cancers compared with previous generations (1,2). Analysis of Surveillance, Epidemiology and End results data from 1975-2015 reveals a striking divergence: While colon and rectal cancer incidence rose by 143 and 111%, respectively, among AYAs aged 15-39 years, rates in older populations declined over the same period (Fig. 1). This divergence in incidence trends between AYAs and older adults is illustrated in Fig. 1, supporting the need to consider age-specific mechanisms rather than attributing the trend solely to enhanced detection. Of note, each incremental unit of accelerated biological aging has been found to be correlated with a 22% increase in cancer risk (2,3), suggesting that biological rather than chronological age may contribute to tumorigenesis in AYAs aged 15-39 years. Whether this reflects earlier exposure to risk factors or distinct biological mechanisms remains unclear.

This phenomenon cannot be attributed solely to enhanced detection or screening practices. However, it should be acknowledged that changes in surveillance, diagnostic practices and awareness are likely contributing to the observed trends to some degree. Rather, accumulating evidence suggests that premature immunosenescence, the early onset of immune system decline typically associated with advanced age, may be a contributing pathogenic mechanism (4-6). Unlike elderly populations where immunosenescence evolves gradually over decades, AYA individuals may experience compressed immune aging driven by metabolic stress, environmental exposures, and possibly epigenetic factors. Whether this truly represents ‘aging’ in the biological sense, or rather a distinct pathological state, remains a matter of debate and is discussed further below.

The tumor microenvironment (TME) in AYA GI cancers may exhibit distinct immunological features, as compared with conventional age-matched or elderly counterparts. Genetic mechanisms of immune evasion in colorectal cancer involve coordinated alterations in antigen presentation, interferon signaling, and oncogenic pathways that converge to suppress antitumor immunity (7). While extensive literature has characterized age-associated immunosenescence in geriatric populations, the mechanisms governing premature immune aging in this population, and its specific interplay with GI TME remodeling, remain surprisingly poorly defined. This knowledge gap has profound therapeutic implications, as AYA patients often receive standardized protocols designed for older populations, potentially overlooking age-specific vulnerabilities and resistance mechanisms.

In the present systematic review, an attempt was made to synthesize available evidence into a coherent framework. The metabolic-inflammatory-hormonal (M-I-H) axis was proposed as a working model to explore how diverse etiological drivers might converge on immune dysregulation in AYA GI cancers. It needs to be emphasized from the outset that this framework remains largely hypothetical, requiring validation through prospective studies specifically designed for AYA populations. Throughout the present systematic review, the strength of evidence supporting each mechanistic claim is annotated to distinguish established findings from speculative extensions.

## Data and methods

A comprehensive literature search of PubMed/MEDLINE (https://pubmed.ncbi.nlm.nih.gov/) between January 1990 and December 2024 was conducted. The search strategy employed combinations of keywords and controlled vocabulary (MeSH terms) related to young-onset GI cancer, immunosenescence and the TME.

### Search strategy

Representative search strings included the following Boolean combinations: (‘young adult’ OR ‘adolescent’ OR ‘early-onset’ OR ‘AYA’) AND (‘GI cancer’ OR ‘colorectal cancer’ OR ‘gastric cancer’ OR ‘esophageal cancer’ OR ‘pancreatic cancer’) AND (‘immunosenescence’ OR ‘immune aging’ OR ‘T cell exhaustion’ OR ‘senescence-associated secretory phenotype’ OR ‘SASP’) for the immunosenescence-focused queries, and (‘metabolic reprogramming’ OR ‘cholesterol metabolism’ OR ‘lactate’ OR ‘leptin’ OR ‘insulin resistance’ OR ‘cortisol’ OR ‘estrogen’ OR ‘microbiome’ OR ‘microbiota’) AND (‘tumor microenvironment’ OR ‘immunosuppression’ OR ‘regulatory T cells’ OR ‘myeloid-derived suppressor cells’) AND (‘GI’ OR ‘colorectal’ OR ‘gastric’) for the mechanism-focused queries. MeSH terms included ‘Neoplasms by Age Group/young adult’, ‘Immunosenescence’, ‘Tumor Microenvironment’, and ‘Gastrointestinal Neoplasms’. The reference lists of included articles were further searched through citation tracking to identify additional relevant studies.

### Selection criteria

Studies were included if they: i) Were published in English; ii) reported on human subjects, preclinical models or mechanistic *in vitro* studies relevant to GI cancer and/or immunosenescence; iii) addressed AYA populations (15-39 years) or provided age-stratified data; or iv) provided foundational mechanistic insights applicable to the M-I-H framework. Studies were excluded if they: i) were case reports with <5 patients; ii) were non-peer-reviewed sources or conference abstracts without full text; iii) focused exclusively on pediatric (<15 years) or elderly (>65 years) populations without relevance to AYA pathophysiology; or iv) were duplicate publications.

### Criteria for high-impact study selection

In this systematic review, studies meeting at least one of the following criteria were prioritized: i) Published in high-impact journals (impact factor >10 or top quartile in the relevant field); ii) large cohort studies (n>1,000 for epidemiological studies) or mechanistic studies with robust experimental design; iii) systematic reviews and meta-analyses; or iv) seminal studies frequently cited (>100 citations) in the field. When overlapping systematic reviews or large consortia data were identified, the most comprehensive or most recent publication was cited to reduce the risk of double-counting evidence.

### Evidence grading

To distinguish established findings from speculative extensions, mechanistic claims were annotated throughout the manuscript with evidence strength labels: i) Evidence robust, for findings supported by multiple human studies or large cohorts; ii) evidence moderate, for findings supported by limited human data or strong preclinical evidence; iii) evidence preclinical only, for findings from animal or *in vitro* models; and iv) evidence hypothesized extension for AYAs, for extrapolations to AYA GI cancer populations where direct evidence was lacking.

### PRISMA screening

**Figure 2:**
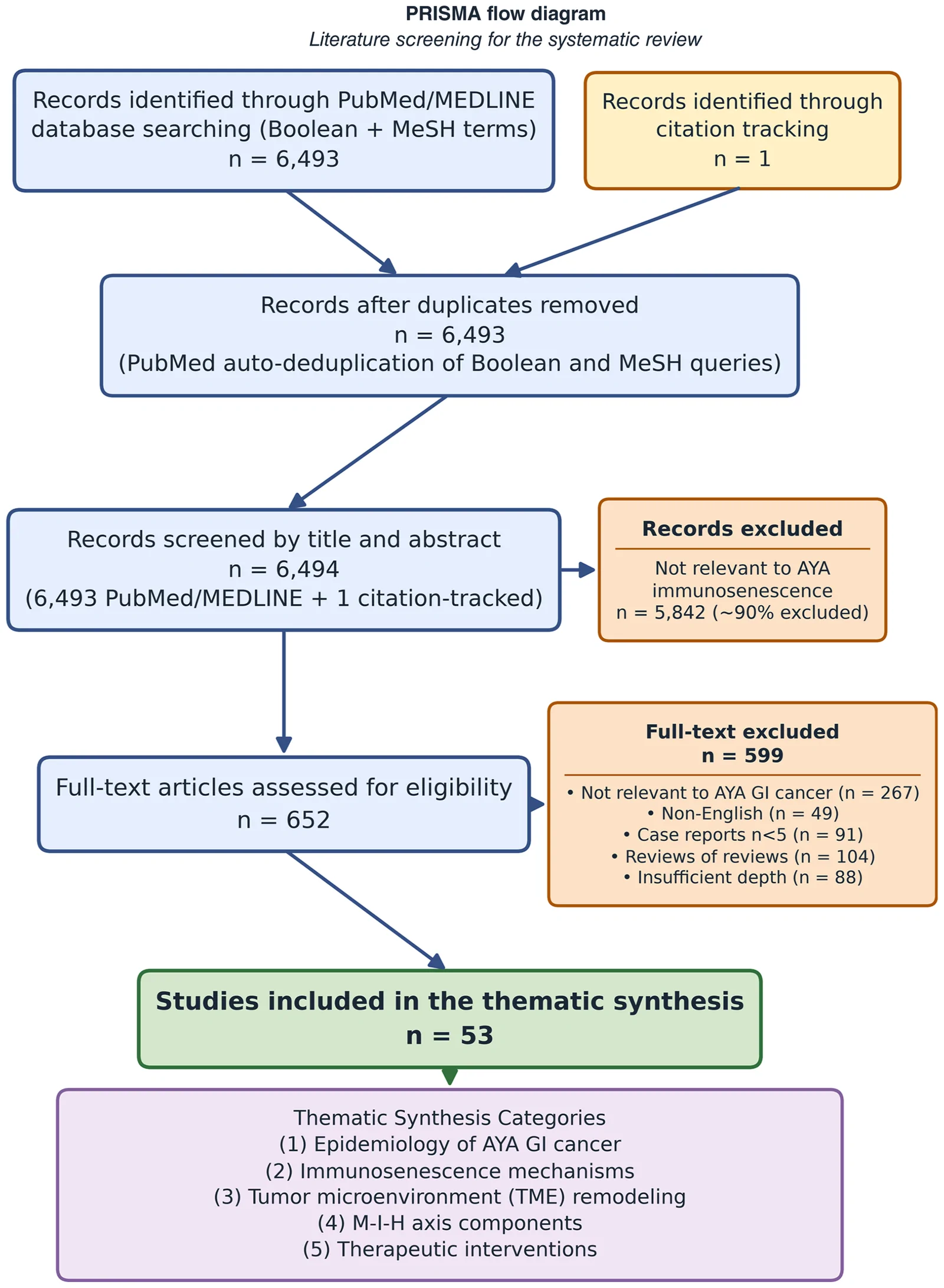
PRISMA flow diagram of literature screening. Records identified through PubMed/MEDLINE database searching (n=6,493) were combined with citation-tracked records (n=1) and screened by title and abstract (n=6,494), assessed for full-text eligibility (n=652), and included in the thematic synthesis (n=53). Embase was not searched due to institutional access limitations. AYA, adolescents and young adults.

A flow diagram summarizing the screening process was provided (Fig. 2). Briefly, the search identified 6,493 unique records through PubMed/MEDLINE (following PubMed auto-deduplication of Boolean keyword queries and MeSH term searches). An additional 1 record was identified through citation tracking (a conference proceedings abstract), yielding 6,494 records screened by title and abstract. Of these, 652 full-text articles were assessed for eligibility, and 53 studies were included in the thematic synthesis. It should be noted that Embase (https://www.embase.com/) was not searched due to institutional access limitations, which is acknowledged as a limitation of this systematic review.

In the present systematic review, current evidence was synthesized to propose the M-I-H axis without performing a meta-analysis. It is explicitly acknowledged that this approach is subject to selection bias and that the conclusions represent the authors' interpretation of the available literature.

## Results

### Molecular mechanisms of premature immunosenescence

Immunosenescence encompasses progressive immune system deterioration characterized by reduced naïve T cell (Tn) output, accumulation of exhausted and senescent memory populations, chronic low-grade inflammation (‘inflammaging’), and metabolic inflexibility (8). In young adults, these processes may be accelerated through distinct molecular pathways that compromise antitumor surveillance.

### Operational definition of premature immunosenescence

A critical challenge in studying premature immunosenescence is the lack of a standardized, operational definition. Canonical age-related immunosenescence is well characterized by thymic involution, accumulation of cluster of differentiation (CD)28^-^ CD57^+^ senescent T cells, declining T-cell receptor excision circle (TREC) levels, telomere shortening and chronic low-grade inflammation (6,8). Premature immunosenescence in AYAs may share these hallmarks, but is likely to differ in tempo, triggers and qualitative phenotype.

It was proposed herein that premature immunosenescence in the AYA context be defined as the presence of accelerated immune aging biomarkers, normally observed in much older individuals, yet appearing in those aged 15-39 years, in the absence of known accelerated aging syndromes. Specifically, the following measurable biomarker panel is suggested for future clinical studies:

i) CD28^-^ CD57^+^ senescent T-cell expansion: A hallmark of T-cell senescence. In healthy elderly individuals, these cells comprise 15-20% of the CD8^+^ T-cell pool; levels above this threshold in AYAs would suggest premature senescence (evidence robust in elderly populations; hypothesized extension for AYAs) (6,9);

ii) TREC levels: As a measure of thymic output, TRECs decline with age. AYA patients with TREC levels below the 10th percentile for their age group may exhibit premature thymic involution (evidence robust as thymic output marker; hypothesized extension for AYA cancer patients);

iii) p16ᴸᴺ_4_ᵃ expression in peripheral blood mononuclear cells: p16ᴸᴺ_4_ᵃ is a robust marker of cellular senescence. An elevated expression in AYA patients would indicate accelerated senescent cell burden (evidence moderate in aging research; hypothesized extension for AYAs);

iv) telomere length in leukocytes: Shortened telomeres in AYA cancer patients compared with age-matched healthy controls would support the premature aging hypothesis (evidence robust in aging research; limited data in AYA cancer);

v) epigenetic aging indices (such as Horvath clock and PhenoAge): These DNA methylation-based biomarkers estimate biological age. An epigenetic age acceleration of >5 years in AYA cancer patients would be consistent with premature immunosenescence (evidence robust in aging research; emerging data in cancer populations).

An important distinction must be made between T-cell exhaustion and T-cell senescence, as these represent molecularly and functionally distinct states. T-cell exhaustion is a progressive, potentially reversible loss of effector function driven by chronic antigen stimulation, characterized by the expression of inhibitory receptors [programmed cell death protein 1 (PD-1), T-cell immunoglobulin and mucin-domain containing-3 (TIM-3) and lymphocyte-activation gene 3] and a hierarchical loss of cytokine production (10,11). T-cell senescence, by contrast, is a stable state of cell-cycle arrest characterized by CD28 loss, CD57 acquisition, telomere shortening, p16ᴸᴺ_4_ᵃ/p21 upregulation and production of the senescence-associated secretory phenotype (SASP). While molecularly distinct, these states may coexist and potentiate each other: Chronically exhausted T cells may acquire senescent features over time, and senescent cells may further suppress remaining functional T cells through SASP-mediated paracrine effects. In the AYA cancer context, both states may be prematurely induced, and distinguishing between them has therapeutic implications, as exhaustion is partially reversible with checkpoint blockade, whereas senescence may require senolytic approaches.

In summary, premature immunosenescence in AYAs may represent either an earlier onset of canonical aging hallmarks or a distinct qualitative phenotype. The biomarker panel proposed above is intended to operationalize this concept for future clinical studies, though it is emphasized that validated thresholds for AYA populations do not yet exist.

### T-cell dysfunction: The core defect

T cells constitute the primary effectors of antitumor immunity; their functional erosion represents the cardinal feature of immunosenescence.

*Exhaustion marker co-expression*. Aged individuals demonstrate progressive accumulation of PD-1^+^ TIM-3^+^ CD8^+^ T cells, phenotypically exhausted populations with severely diminished cytotoxic capacity (6,12). In murine models, these cells markedly expand by 65 weeks (human equivalent: 50-58 years), coinciding with Tn depletion and effector memory T-cell predominance (12) (evidence robust in murine models). In young cancer patients, chronic antigen stimulation, driven by dysbiotic microbiota, metabolic stress or subclinical infections, may compress this timeline, inducing premature exhaustion signatures detectable in peripheral blood (evidence hypothesized extension for AYAs).

*Thymic involution*. Post-pubertal thymic atrophy reduces Tn output and T-cell receptor (TCR) diversity, constraining the repertoire available for neoantigen recognition (13). Quantitatively, healthy young adults maintain ~271 Tns/µl (CD45RA^+^CCR7^+^), declining to 226 in elderly individuals, while memory T cells increase from 419 to 472/µl (14) (evidence robust in elderly populations). Thymic function profiles in young cancer patients are often observed to fall between those of healthy peers and elderly controls, suggesting accelerated aging (evidence moderate; hypothesized extension for AYAs). Recent data have indicated that thymic stromal lymphopoietin and IL-7 signaling, which are critical for T-cell homeostasis, are suppressed in obesity and metabolic syndrome, conditions increasingly prevalent among young adults (15) (evidence robust for metabolic-immune link; hypothesized extension for AYA GI cancer).

*Co-stimulatory deficiency*. CD28 expression on peripheral T cells decreases significantly with immune aging (49.64±8.55% vs. 39.17±10.19% in the elderly; P=0.012), impairing second-signal transduction and exacerbating functional exhaustion (9) (evidence robust in elderly populations). This downregulation may occur prematurely in the context of chronic metabolic inflammation, creating ‘pseudo-aged’ T-cell phenotypes in young hosts (evidence hypothesized extension for AYAs).

### Inflammatory reprogramming: Beyond simple inflammation

Chronic inflammation promotes tumorigenesis through multiple pathways, but the specific inflammatory signature of premature immunosenescence may differ from conventional inflammaging.

*The SASP conundrum*. Senescent cells secrete IL-1α/β, TNF-α, IL-6 and proteases, collectively termed the SASP (16) (evidence robust). Park *et al* (17) recently demonstrated that IL-1α signaling accelerates cancer progression through emergency myelopoiesis (evidence robust in preclinical models), suggesting a direct mechanistic link. In young adults, obesity-driven SASP activation from adipose tissue might similarly promote GI carcinogenesis, although the relative contribution of adipose vs. tumor-derived SASP factors remains unclear (evidence: It is increasingly recognized that senescent cells do not exhibit uniform SASP profiles, and the tissue-specific consequences of SASP activation in young patients represent an active area of investigation).

*Extracellular vesicles: An emerging paradigm*. Tumor-derived extracellular vesicles (tEVs) carrying programmed death-ligand 1 (PD-L1) and microRNAs can induce T-cell senescence-like states through cAMP response element-binding protein/signal transducer and activator of transcription (STAT) activation (18) (evidence preclinical only). The clinical significance of this observation remains uncertain, extracellular vesicle (EV) concentrations in young vs. elderly cancer patients have not been systematically compared, and whether EV-mediated suppression differs qualitatively between age groups is unknown. To the best of our knowledge, no published evidence directly compares tEV prevalence or functional impact in AYA GI cancer TME vs. older patients. Nevertheless, the possibility that tEVs create systemic immunosuppressive fields, enabling pre-metastatic niche formation, suggests a therapeutic potential for EV biogenesis inhibitors. However, GW4869, the most commonly studied neutral sphingomyelinase-2 (nSMase2) inhibitor, is a research tool with poor pharmacokinetic properties and limited *in vivo* bioavailability, making it unsuitable for clinical translation (19). Next-generation nSMase2 inhibitors with improved drug-like properties are needed before EV-targeted strategies can be tested clinically in AYA GI cancers (evidence preclinical only; no clinical trials in AYA populations).

### Metabolic competition within the TME

The metabolic landscape of tumors fundamentally shapes immune responses, with implications for the efficacy of immunotherapy.

*Lipid metabolism dysregulation*. Tumor cells outcompete T cells for glucose and fatty acids through aerobic glycolysis (Warburg effect), inducing effector cell energy depletion. The upregulation of 3-hydroxy-3-methylglutaryl-CoA reductase (HMGCR), the rate-limiting enzyme in cholesterol synthesis, promotes lipid droplet accumulation in T cells, mechanically stabilizing PD-1 expression and reinforcing exhaustion independently of ligand engagement (20,21) (evidence robust in preclinical models; hypothesized extension for AYAs). This ‘metabolic checkpoint’ offers a complementary therapeutic target to conventional immune checkpoint blockade. However, direct evidence of HMGCR upregulation within the TME of AYA GI cancer, relative to GI cancer in older patients, is currently lacking and constitutes a critical knowledge gap.

*Lactate accumulation*. Tumor-derived lactate acidifies the microenvironment (pH 6.5-6.8), suppressing CD8^+^ T-cell proliferation and interferon-γ production while selectively expanding immunosuppressive regulatory T cells (Tregs) through hydroxycarboxylic acid receptor 1 (HCAR1) signaling (22,23) (evidence robust in preclinical models). The PD-1/PD-L1 axis serves as a master regulator of T-cell exhaustion, with co-expression of multiple inhibitory receptors marking terminally dysfunctional states refractory to reactivation (10). HCAR1 actively recruits polymorphonuclear myeloid-derived suppressor cells (MDSCs), establishing a feed-forward immunosuppressive loop (22) (evidence robust in colorectal cancer preclinical models). Young patients with high glycolytic tumors (as indicated by fluorodeoxyglucose positron emission tomography avidity) may exhibit particularly pronounced lactate-mediated immune dysfunction (evidence hypothesized extension for AYAs). Pan-cancer analyses further demonstrate that lactate metabolism signatures predict immunotherapy response and survival outcomes, underscoring the clinical utility of metabolic profiling in this population (24) (evidence robust in pan-cancer cohorts).

In summary, the molecular mechanisms of premature immunosenescence, T-cell dysfunction, inflammatory reprogramming and metabolic competition, are well established in elderly and preclinical contexts but remain largely hypothetical when extended to AYA GI cancer. Direct evidence from age-stratified comparisons is urgently needed.

### Tumor microenvironment remodeling in young-onset disease

The TME represents a dynamic ecosystem where malignant cells co-opt normal physiological processes to evade immune destruction (25,26). In AYA GI cancers, TME features may reflect the imprint of premature immunosenescence, though direct comparative studies between young and elderly TMEs are limited.

### Immunosuppressive cell infiltration. Tregs

Forkhead box P3 (FOXP3)^+^ Tregs accumulate within GI tumors, secreting IL-10 and TGF-β to suppress CD8^+^ cytotoxic function. High intratumoral Treg density is correlated with advanced stage and poor prognosis in colorectal cancer (27-29) (evidence robust in colorectal cancer). Of note, intratumoral Tregs may originate from converted Th1 cells, with CD39-mediated adenosinergic signaling representing a key suppressive mechanism distinct from cytokine-dependent inhibition (30) (evidence robust in preclinical models). Young patients may exhibit particularly Treg-dominated microenvironments due to hormonal influences (estrogen-enhanced Treg stability) and metabolic factors (leptin-driven Treg expansion) (evidence hypothesized extension for AYAs).

*MDSCs*. MDSCs consume essential amino acids (arginine and cysteine) through arginase-1 and inducible nitric oxide synthase, starving T cells while producing reactive oxygen and nitrogen species (31) (evidence robust). Elevated circulating MDSCs predict chemotherapy resistance in gastric cancer (32) (evidence robust in gastric cancer), representing a potential biomarker for treatment stratification in young adults (evidence hypothesized extension for AYAs).

*Tumor-associated macrophages (TAMs)*. M2-polarized TAMs dominate the GI TME, secreting IL-6 and IL-10 to promote angiogenesis and upregulate PD-L1 expression on tumor cells (33,34) (evidence robust). These cells originate from both tissue-resident populations and recruited monocytes, with their polarization state influenced by lactate and lipid metabolites abundant in metabolically dysregulated young patients (evidence robust for metabolic polarization; hypothesized extension for AYAs). Beyond macrophage polarization, IL-6 critically mediates tumor-stromal cross-talk, particularly with activated fibroblasts, to establish immunosuppressive niches (35) (evidence robust in esophageal cancer).

### Metabolic and hypoxic barriers

Beyond lactate-mediated suppression, the TME imposes additional metabolic constraints. Hypoxia-inducible factor-1α (HIF-1α) activation upregulates vascular endothelial growth factor and PD-L1, simultaneously promoting vascular dysfunction and adaptive immune resistance (36) (evidence robust). Hypoxic regions are poorly penetrable by T cells, creating sanctuaries for resistant tumor clones. Targeting HIF-1α or improving vascular normalization may enhance the efficacy of immunotherapy.

### Gut microbiota interactions

The intestinal microbiome critically modulates antitumor immunity and represents a key interface between diet, metabolism and the immune system. Microbial fermentation products, particularly butyrate, enhance CD8^+^ T-cell function through the inhibition of histone deacetylase (HDAC) (37) (evidence robust). The gut microbiome modulates host immunity through diverse mechanisms, including the production of immunomodulatory metabolites, regulation of systemic inflammatory tone and modulation of therapeutic responses (38) (evidence robust). Conversely, dysbiosis-associated depletion of short-chain fatty acids (SCFAs) and expansion of pro-inflammatory species [such as *Fusobacterium nucleatum (F. nucleatum*)] compromise immune surveillance (39) (evidence robust in colorectal cancer). *F. nucleatum* specifically promotes chemoresistance in colorectal cancer through Toll-like receptor 4 (TLR4)/myeloid differentiation primary response 88 (MyD88) activation and autophagy modulation. The mechanistic integration of the microbiome within the M-I-H axis are discussed below in detail.

### Metabolic-inflammatory-hormonal axis

**Figure 3:**
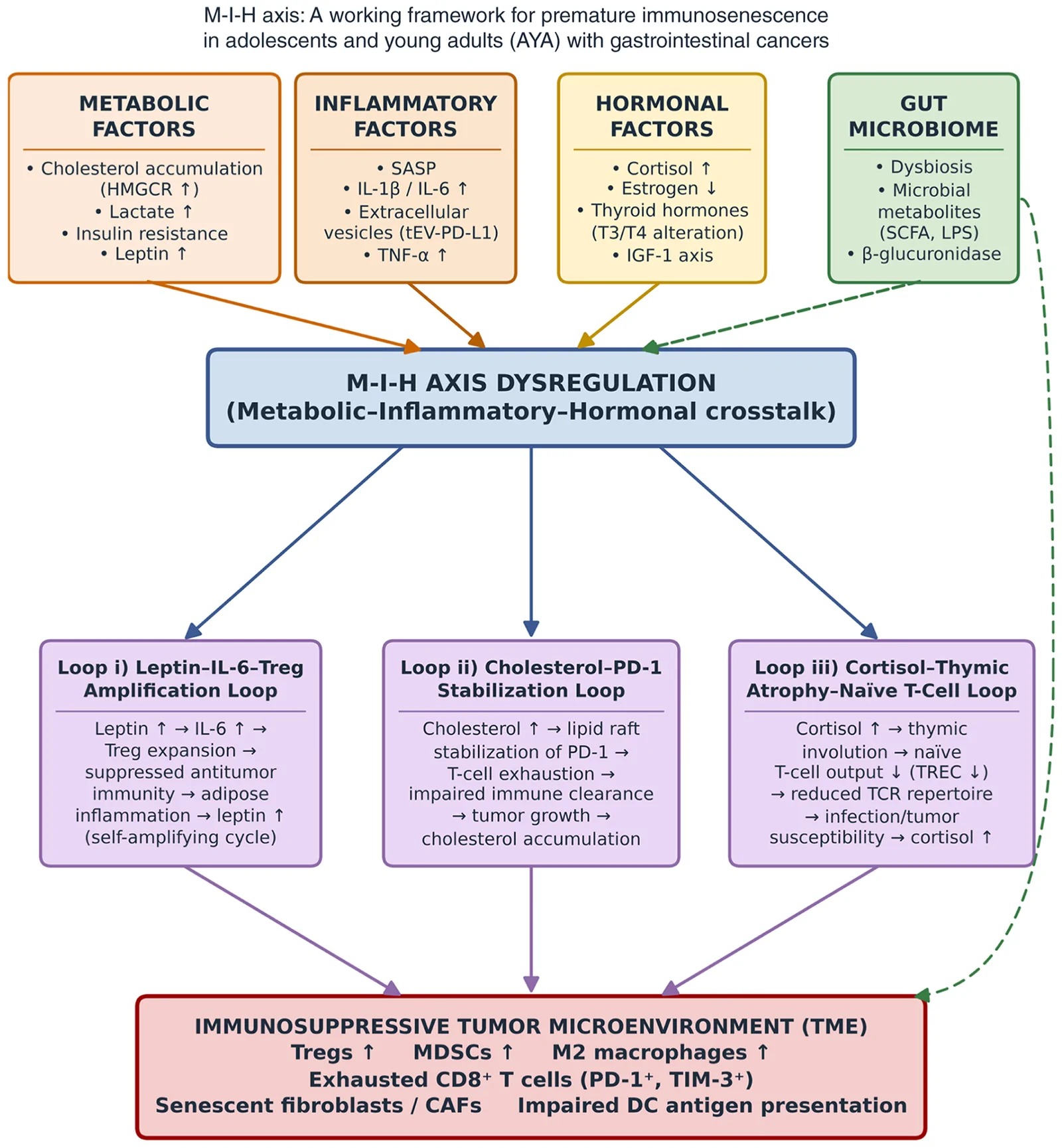
M-I-H axis as a working framework for premature immunosenescence in AYA gastrointestinal cancers. The schematic illustrates how metabolic factors (cholesterol accumulation, lactate, insulin resistance, and leptin), inflammatory factors (SASP, IL-1β/IL-6, and extracellular vesicles), hormonal factors (cortisol, estrogen, and thyroid hormones), and the gut microbiome interact through three key feedback loops: i) The leptin-IL-6-Treg amplification loop; ii) the cholesterol-PD-1 stabilization loop; and iii) the cortisol-thymic atrophy-naïve T-cell depletion loop. These interconnected pathways converge to create an immunosuppressive TME enriched with Tregs, MDSCs, and M2 macrophages. It is emphasize that this model is a working hypothesis; the dashed arrows indicate hypothesized pathways requiring direct experimental validation in AYA populations. M-I-H, metabolic-inflammatory-hormonal; AYA, adolescents and young adults; HMGCR, 3-hydroxy-3-methylglutaryl-CoA reductase; HCAR1, hydroxycarboxylic acid receptor 1; HIF-1α, hypoxia-inducible factor-1α; MDSCs, myeloid-derived suppressor cells; SASP, senescence-associated secretory phenotype; TME, tumor microenvironment; Tregs, regulatory T cells; TSLP, thymic stromal lymphopoietin.

The M-I-H axis is proposed as an integrative framework for exploring how diverse etiological factors might converge on immune dysregulation in AYA GI cancers (Fig. 3). A schematic of the M-I-H axis, illustrating how metabolic, inflammatory, hormonal, and microbial factors interact through feedback loops to create an immunosuppressive TME is presented in Fig. 3. It is emphasized that this model remains hypothetical, requiring direct experimental validation in age-stratified cohorts. For each component, the hypothesized pathway leading from dysregulation to a specific immunosuppressive effect is stated.

### Steroid hormone imbalance. Cortisol and chronic stress

Modern lifestyles impose chronic psychosocial stress, elevating cortisol levels. It is hypothesized that chronic glucocorticoid signaling induces T-cell exhaustion and thymic atrophy (40) (evidence robust for mechanism), potentially accelerating immune aging in young adults through the following pathway: Elevated cortisol → glucocorticoid receptor activation in thymic epithelial cells → accelerated thymic involution → reduced Tn output and TCR diversity → impaired neoantigen recognition → diminished tumor immune surveillance (evidence hypothesized extension for AYAs). The clinical significance of stress-induced immunosuppression in cancer progression remains difficult to quantify, though mechanistic plausibility is strong.

*Sex hormones and immune aging*. Estrogen receptor signaling accelerates thymic involution (15) (evidence robust in preclinical models), while selective estrogen receptor modulators (SERMs) can restore T-cell populations in models (41) (evidence preclinical only). The observed male predominance in young-onset colorectal cancer may partly reflect androgen-mediated immune effects, though disentangling hormonal from behavioral risk factors remains challenging.

A more nuanced analysis suggests that the protective effects of estrogen may be more important than male-specific risk factors. Estrogen maintains intestinal barrier integrity, modulates gut microbiome composition and suppresses pro-inflammatory cytokine production through estrogen receptor β signaling in intestinal epithelial cells and immune cells (26) (evidence robust for mechanism). The lower baseline incidence of colorectal cancer in premenopausal women may reflect these estrogen-mediated protective mechanisms. Furthermore, the declining age at menarche observed globally may alter lifetime estrogen exposure patterns, potentially impacting the window of immune protection. Oral contraceptive use, prevalent among AYA women, alters hormonal profiles and may modulate cancer risk through mechanisms not yet fully understood. Whether the protective effects of estrogen are more important than male-specific risk factors remains an open question warranting dedicated investigation (evidence hypothesized; requires age- and sex-stratified studies).

### Insulin-leptin-thyroid axis. Insulin resistance

It is hypothesized that the following pathway exists: Hyperinsulinemia → phosphoinositide 3-kinase/protein kinase B/mechanistic target of rapamycin activation in tumor-related macrophages → PD-1 upregulation on macrophages → T-cell suppression in the TME (42,43) (evidence robust in preclinical models; hypothesized extension for AYAs). Given rising obesity rates among young adults, this mechanism may be increasingly relevant.

*Leptin signaling*. It is hypothesized that in the context of obesity-associated hyperleptinemia, Janus kinase 2/STAT3 signaling in the TME preferentially expands Treg populations over effector T cells, thereby contributing to the immunosuppressive microenvironment characteristic of AYA GI cancers (44) (evidence robust for mechanism in preclinical models; hypothesized extension for AYAs). Leptin receptor antagonists improve checkpoint inhibitor responses in preclinical models (45) (evidence preclinical only), although human trials are lacking. Whether leptin levels stratify immunotherapy response in young, obese patients represents a testable hypothesis. Elevated soluble leptin receptor levels have been associated with advanced tumor stage in patients with colorectal cancer (45) (evidence moderate in colorectal cancer), providing preliminary support for this hypothesis.

*Thyroid hormones*. It is hypothesized that altered thyroid hormone status may drive metabolic exhaustion in T cells through the following pathway: T3/T4 enhance T-cell glycolysis while impairing oxidative phosphorylation (11,46) → metabolic exhaustion of effector T cells → reduced anti-tumor cytotoxicity (evidence preclinical only; hypothesized extension for AYAs). Subclinical hypothyroidism, frequently undiagnosed, might modulate cancer immunosurveillance, although direct evidence is limited.

### Cholesterol metabolism and immune regulation

The HMGCR/sterol O-acyltransferase 2 axis links lipid metabolism to immune suppression (21) (evidence robust in preclinical models). In addition, the inhibition of cholesterol biosynthesis promotes antitumor immunity through the suppression of long non-coding RNA SNHG29-mediated activation of Yes-associated protein (YAP), revealing an epigenetic layer of regulation within metabolic-immune crosstalk (47) (evidence preclinical only).

Regarding the clinical evidence for statins, a nationwide cohort study suggested an association between statin use and reduced gastric cancer incidence and mortality (48) (evidence moderate; observational data subject to residual confounding). Mechanistically, targeting the mevalonate pathway can potentiate NUA kinase 1 inhibition-induced immunogenic cell death, linking metabolic intervention directly to enhanced antitumor immunity (49) (evidence preclinical only). However, the statin-cancer association is based primarily on observational studies and is not universally accepted; conflicting evidence exists, and the possibility of healthy-user bias cannot be excluded. Whether young patients with dyslipidemia represent a particularly responsive subgroup requires prospective evaluation in randomized controlled trials.

### Gut microbiome: The metabolic-inflammatory interface

The gut microbiome serves as a critical mechanistic bridge within the M-I-H axis, linking dietary inputs to metabolic and inflammatory outputs that directly shape the TME. Rather than functioning merely as a therapeutic target, the microbiome is an integral component of the axis whose dysregulation amplifies immunosenescence.

The following pathway is hypothesized: High-fat Western diet → altered bile acid metabolism → increased secondary bile acids (deoxycholic acid, lithocholic acid) → activation of farnesoid X receptor and takeda G-protein-coupled receptor 5 signaling in intestinal epithelium → enhanced pro-inflammatory cytokine production and SASP activation → immunosuppressive TME remodeling (evidence robust for bile acid-immune link in preclinical models; hypothesized extension for AYAs). Secondary bile acids represent a direct mechanistic link between the metabolic and inflammatory arms of the M-I-H axis.

Additional microbial metabolite pathways include: i) Tryptophan derivatives (indole-3-acetic acid, kynurenine) acting as aryl hydrocarbon receptor ligands that modulate IL-22 production and maintain mucosal barrier integrity (38) (evidence robust); ii) SCFAs (butyrate, propionate, acetate) serving as HDAC inhibitors that enhance CD8^+^ T-cell function while promoting Treg differentiation in a context-dependent manner (37) (evidence robust); and iii) the feed-forward loop whereby dysbiosis promotes metabolic dysregulation (such as reduced SCFA production impairs intestinal barrier function, increasing systemic endotoxemia and metabolic inflammation), which in turn further disrupts microbiome composition (evidence moderate; hypothesized extension for AYA GI cancer).

*F. nucleatum* exemplifies how specific dysbiosis-associated organisms integrate into the M-I-H axis: Through TLR4/MyD88 activation, it amplifies local inflammatory signaling, while simultaneously promoting autophagy-driven chemoresistance (39) (evidence robust in colorectal cancer). This creates an inflammatory-metabolic feedback loop that may be particularly relevant in young patients whose microbiome composition has been disrupted by antibiotic exposure, dietary changes or obesity.

### Axis crosstalk and feedback mechanisms

A key limitation of the initial framework was the treatment of metabolic, inflammatory, and hormonal components as separate entities. In reality, these axes are deeply interconnected through specific crosstalk pathways and feedback loops:

*Metabolic-inflammatory crosstalk*. Cholesterol accumulation in immune cells activates the NLRP3 inflammasome, amplifying IL-1β and IL-6 production. This creates a feed-forward loop: Metabolic dysregulation → inflammasome activation → cytokine release → further metabolic reprogramming of immune cells (20,21) (evidence robust in preclinical models). Conversely, lactate accumulation stabilizes HIF-1α, which upregulates PD-L1 and promotes M2 macrophage polarization, linking metabolic and immune evasion pathways.

*Inflammatory-hormonal crosstalk*. Chronic IL-6 elevation disrupts the hypothalamic-pituitary-adrenal (HPA) axis, altering cortisol circadian rhythms. This creates a vicious cycle: Chronic inflammation → HPA dysregulation → altered cortisol patterns → impaired immune regulation → further inflammation (evidence moderate; hypothesized extension for AYAs). In addition, IL-6 and TNF-α modulate sex hormone binding globulin (SHBG) production, affecting free estrogen and testosterone levels (35,44).

*Hormonal-metabolic crosstalk*. Insulin resistance and hyperleptinemia suppress SHBG production, increasing free estradiol in obese young women (42,44). This links the metabolic and hormonal arms: Obesity → hyperinsulinemia → reduced SHBG → elevated free estrogen → ER-signaling-driven thymic involution and Treg expansion (evidence moderate; hypothesized extension for AYAs).

Three key feedback loops merit particular attention: i) The leptin-IL-6-Treg amplification loop, where leptin-driven IL-6 production expands Tregs, which in turn suppress antitumor immunity and promote tumor growth, further increasing leptin production through cancer-associated cachexia and adipose tissue remodeling; ii) the cholesterol-PD-1 stabilization loop, where HMGCR-driven cholesterol accumulation stabilizes PD-1 expression, leading to T-cell exhaustion and reduced immune-mediated tumor clearance, perpetuating the metabolic TME; and iii) the cortisol-thymic atrophy-Tn depletion loop, where chronic stress elevates cortisol, accelerating thymic involution and reducing Tn output, further compromising immune surveillance and allowing tumor progression that amplifies inflammatory stress (evidence hypothesized for AYAs; requires prospective validation).

### GI cancer specificity: Why the digestive tract?

A critical question is why GI cancers, in particular, may be susceptible to M-I-H-driven immunosenescence. Several anatomical and functional features make the digestive tract uniquely vulnerable:

First, the direct anatomical interface between the gut microbiome and the intestinal immune system, the largest lymphoid organ in the body, means that microbiome dysregulation has immediate and profound effects on local and systemic immunity. The gut-associated lymphoid tissue contains ~70% of the immune cells of the body, making it particularly susceptible to premature senescence driven by microbiome-mediated inflammatory and metabolic signals (38) (evidence robust for anatomy; hypothesized for premature senescence).

Secondly, the metabolic roles of the liver and pancreas as integral GI organs create a metabolic-immune nexus unique to the digestive system. The liver, as the primary site of cholesterol and bile acid metabolism, is directly exposed to gut-derived microbial products through the portal circulation (37,38). Pancreatic hormones (insulin and glucagon) regulate systemic metabolism and influence immune cell function. Dysfunction in these organs amplifies the metabolic arm of the M-I-H axis (evidence moderate; hypothesized for AYA specificity).

Thirdly, the GI tract is continuously exposed to dietary carcinogens, environmental toxins and microbial products that may accelerate immune aging through chronic antigenic stimulation and inflammatory signaling (38,39). Young adults with evolving dietary patterns (processed foods and high-fat diets) may experience particularly intense exposure to microbiome-disrupting and pro-inflammatory dietary components (evidence moderate for dietary impact on microbiome; hypothesized for AYA immunosenescence).

Fourth, the high baseline immune activity of the intestinal mucosa, maintaining tolerance to commensal organisms while defending against pathogens (38), may render this compartment especially vulnerable to premature exhaustion and senescence. Unlike other tissues where immune activity is primarily reactive, the gut immune system is constitutively active, potentially accelerating immune cell turnover and senescent cell accumulation (evidence hypothesized; requires investigation).

In summary, the M-I-H axis provides a unifying framework that is particularly applicable to GI cancers due to the unique anatomical and functional characteristics of the digestive system. However, the specific predictions of this framework, particularly regarding AYA-specific mechanisms, remain to be assessed empirically.

## Discussion

### Therapeutic implications and future directions

The M-I-H axis framework suggests multiple intervention points (Table I). However, it should be noted that that most available evidence is derived from preclinical models or elderly populations, with limited data generated specifically from AYA GI cancer patients. Interventions are prioritized by clinical readiness, strength of evidence and mechanistic alignment with the M-I-H axis.

### Priority cluster 1: Metabolic modulation combined with immunotherapy

Repurposing widely available metabolic agents (statins and metformin) in combination with immune checkpoint inhibitors represents the most immediately testable strategy. Statins target the HMGCR/cholesterol arm of the M-I-H axis and may have a synergistic effect with anti-PD-1 therapy by destabilizing PD-1 expression on T cells (48,49) (evidence moderate for statin-immunotherapy synergy; preclinical and observational). Metformin addresses the insulin resistance arm and may reduce MDSC infiltration (42) (evidence preclinical and early clinical). However, optimal dosing, timing and patient selection remain undefined, and whether young patients require different approaches than elderly patients has not yet been studied.

### Priority cluster 2: Cytokine and inflammation blockade

The CANTOS trial demonstrated reduced lung cancer incidence with IL-1β inhibition (17) (evidence robust for lung cancer; untested in GI cancer), supporting broader investigation. IL-6 blockade has shown particular promise in esophageal squamous cell carcinoma (50) (evidence moderate). However, long-term anti-IL-6 therapy in young patients carries specific risks, including increased susceptibility to infections, hepatic toxicity and potential interference with normal immune development. The risk-benefit profile of chronic cytokine blockade in AYA patients, who may require decades of treatment, differs substantially from elderly populations and must be carefully evaluated. Whether GI cancers show similar responsiveness to IL-1β blockade, and whether young patients benefit disproportionately, remains unknown.

### Priority cluster 3: Microbiota engineering

Fecal microbiota transplantation and probiotic approaches show promise in melanoma (37) (evidence moderate in melanoma); translation to GI cancers appears logical given anatomical relevance, although technical challenges (donor selection, administration route, and standardization) require resolution. Dietary interventions targeting secondary bile acid production represent a complementary, lower-risk approach that may be particularly suitable for AYA patients.

### AYA-specific considerations

Clinical trial design for M-I-H-targeted interventions in AYAs must address unique considerations: i) Fertility preservation, cytokine blockade and metabolic interventions may impact reproductive function during a critical life stage; ii) long-term toxicity profiles, young patients may live for decades after treatment, making late effects (cardiovascular, metabolic, and secondary malignancies) a primary concern; iii) treatment adherence and psychosocial factors, AYA patients face unique psychosocial challenges that affect treatment compliance and quality of life; and iv) developmental considerations, the immune system may still be maturing in younger AYAs (15-25 years), and interventions targeting immune pathways could have unintended developmental consequences.

### Proposed clinical trial concepts

To operationalize the M-I-H framework, two concrete trial concepts were proposed: i) an AYA-enriched Phase II trial of statin (atorvastatin 40 mg daily) combined with pembrolizumab in MSI-H/dMMR colorectal cancer, stratified by leptin levels and CD28^-^ CD57^+^ T-cell frequency, with primary endpoints of objective response rate and progression-free survival; and ii) a microbiota-stratified AYA cohort receiving standard immunotherapy with pre-specified analysis of microbial metabolite profiles (SCFAs, secondary bile acids) as predictive biomarkers, exploring whether microbiome-targeted dietary interventions improve outcomes.

### Precision oncology and biomarker development

Precision oncology biomarkers can help guide therapy selection, although their specific relationship to the M-I-H axis and premature immunosenescence requires further investigation. It should be noted that the inclusion of these biomarkers in the AYA context currently represents an extrapolation from the broader immuno-oncology field, with limited AYA-specific data available.

### Established biomarkers

Polymerase ε/polymerase δ1 (POLE/POLD1) mutations predict an 89% response rate to PD-1 blockade (51) (evidence robust), and MSI-H/dMMR status is an established predictor of sensitivity to immunotherapy (52) (evidence robust). Whether these molecular subtypes are enriched in AYA GI cancer populations due to premature immunosenescence impairing immune surveillance of mismatch repair-deficient or polymerase-mutant clones is an intriguing but unproven hypothesis. Comprehensive genome and transcriptome signatures provide additional prognostic granularity that may refine patient selection beyond single-gene alterations (53) (evidence robust).

### Emerging biomarkers

TMB may reflect accumulated DNA damage from metabolic and inflammatory stressors central to the M-I-H axis. In this model, chronic inflammation-driven mutagenesis and impaired immune surveillance (due to premature immunosenescence) may collectively elevate TMB in AYA tumors (evidence hypothesized; requires validation in AYA cohorts). Circulating tumor DNA enables minimal residual disease monitoring and may capture real-time immune-tumor interactions modulated by the M-I-H axis, offering a non-invasive window into treatment response (evidence robust in general oncology; limited AYA-specific data). Immune gene signatures, including interferon-γ signatures and T cell-inflamed gene expression profiles, may reflect the specific immune cell composition shaped by premature immunosenescence and could potentially distinguish M-I-H-driven immunosuppression from other immune evasion mechanisms (evidence emerging; hypothesized for AYA specificity).

### M-I-H axis biomarkers

Novel biomarkers specifically aligned with the M-I-H framework, including plasma leptin levels, cortisol circadian rhythm analysis, specific microbiota signatures, and the senescent T-cell biomarker panel previously proposed, may add a predictive value in young patients beyond standard molecular markers. However, these remain research tools without validated clinical thresholds (evidence hypothesized; priority for translational research).

### Evidence synthesis, heterogeneity and knowledge gaps. Established mechanisms vs. hypothesized AYA-specific extensions

To provide transparency about the evidentiary basis of our framework, established mechanisms in GI cancer broadly are explicitly separated from hypothesized AYA-specific extensions within the M-I-H model:

*Established in GI cancer (broadly)*. The following findings are supported by robust evidence across age groups in GI cancer: i) T-cell exhaustion and PD-1/PD-L1 axis involvement in immune evasion (10); ii) Treg accumulation and immunosuppression in the GI TME (27,28); iii) MDSC-mediated T-cell suppression (31); iv) lactate-driven immunosuppression via HCAR1(22); v) *F. nucleatum* promotion of chemoresistance (39); vi) HIF-1α-mediated vascular and immune remodeling (36); and vii) the predictive value of MSI-H/dMMR and POLE/POLD1 for immunotherapy response (51,52).

*Hypothesized AYA-specific extensions*. The following extensions to AYA GI cancer are hypothesized but lack direct experimental evidence: i) premature T-cell exhaustion in AYAs due to compressed immune aging; ii) obesity-driven SASP activation specifically promoting GI carcinogenesis in young adults; iii) leptin-driven preferential Treg expansion in the AYA TME; iv) cortisol-mediated accelerated thymic involution in stressed young adults; v) the M-I-H axis as an integrated framework specifically relevant to AYA GI cancer; and vi) elevated TMB in AYA tumors due to M-I-H-driven mutagenesis. Each of these hypotheses requires testing in age-stratified cohorts.

### Heterogeneity across populations and cancer subtypes

Significant heterogeneity exists across populations and GI cancer subtypes that complicates the M-I-H framework.

*Population variability*. AYA cancer incidence trends differ between Western and Asian populations, with earlier and steeper increases in colorectal cancer incidence observed in the United States, as compared with several Asian countries (3) (evidence robust). Urban vs. rural differences likely reflect variations in dietary patterns, obesity prevalence and healthcare access. These population-level differences suggest that the relative contribution of M-I-H axis components may vary by geographic and cultural context.

*Cancer subtype variability*. Each GI cancer subtype has a distinct immune landscape. Colorectal cancers exhibit diverse immune phenotypes ranging from ‘hot’ (immune-infiltrated, MSI-H) to ‘cold’ (immune-desert). Gastric cancers show variable Epstein-Barr virus-associated and MSI-related immune signatures. Esophageal squamous cell carcinomas differ immunologically from adenocarcinomas. Pancreatic cancers are characteristically immune-desert (7,25). The M-I-H axis may operate differently across these subtypes, and its relevance cannot be assumed to be uniform (evidence robust for subtype differences; hypothesized for M-I-H subtype specificity).

*Study design variability*. The evidence base spans observational epidemiologic studies, preclinical mechanistic studies, and clinical trials, each with different strengths and limitations. Observational studies are subject to confounding; preclinical models may not faithfully reproduce human AYA cancer biology; and clinical trial populations are often enriched for elderly patients, limiting generalizability to AYAs.

### Potential confounders and publication bias

Several potential confounders must be considered when interpreting the evidence for premature immunosenescence in AYA GI cancer:

*Lifestyle factors*. Obesity, dietary patterns, physical activity levels, alcohol consumption, and smoking all influence both cancer risk and immune function (4,43). The rising prevalence of obesity and metabolic syndrome among young adults may independently drive both cancer incidence and immune dysfunction, making it difficult to isolate the specific contribution of premature immunosenescence. Screening practices: Increased awareness and changing diagnostic practices may contribute to apparent incidence increases, although the magnitude of the trend and the presentation at advanced stages argue against detection bias as the sole explanation. Treatment differences: AYA patients may receive different treatment regimens than elderly patients, affecting outcomes and TME characteristics. Antibiotic exposure: Increasing antibiotic use in childhood and adolescence may alter microbiome composition, independently affecting cancer risk and immune function.

*Publication bias*. Positive mechanistic findings, particularly from preclinical studies, are more likely to be published than negative results. This is especially relevant for the M-I-H framework, as many of the proposed mechanisms are based on preclinical data that may not replicate in human AYA populations. The absence of published negative studies may create an overly optimistic impression of the evidence base.

### Limitations and alternative hypotheses

Several limitations of the M-I-H framework are explicitly acknowledged and alternative hypotheses for the rising incidence of AYA GI cancers are discussed.

### Alternative explanation 1: Changes in detection and surveillance

Increased awareness of AYA GI cancer, expanded use of endoscopy and improved imaging techniques may contribute to apparent incidence increases. However, the magnitude of the trend, particularly the presentation of numerous AYA patients at advanced stages, argues against detection bias as the primary driver. Detection changes may account for some of the observed increase but are unlikely to explain the full trend.

### Alternative explanation 2: Obesity and diabetes epidemic

The rising prevalence of obesity and type 2 diabetes among young adults is a well-established risk factor for GI cancers, particularly colorectal cancer. This may operate independently of immunosenescence through insulin-mediated proliferation, adipose tissue inflammation, and altered adipokine profiles. Of note, obesity and metabolic syndrome overlap significantly with the metabolic arm of the M-I-H axis, suggesting that these explanations may be complementary rather than competing.

### Alternative explanation 3: Environmental carcinogens and dietary shifts

Generational shifts in dietary patterns (increased processed foods, red meat, and sugar-sweetened beverages), exposure to environmental toxins (microplastics, food additives, and pesticides), and changes in alcohol consumption patterns may independently increase cancer risk. These factors may interact with the M-I-H axis, for example, dietary changes alter microbiome composition, which in turn modulates immune and metabolic pathways, rather than being mutually exclusive with the immunosenescence hypothesis.

### Alternative explanation 4: Antibiotic exposure and microbiome disruption

Increasing antibiotic use in childhood and adolescence may permanently alter gut microbiome composition, promoting dysbiosis-associated carcinogenesis. This hypothesis is mechanistically linked to the M-I-H axis through the microbiome component, but suggests a different primary driver (iatrogenic microbiome disruption) rather than intrinsic premature immunosenescence.

### Framework limitations

The M-I-H framework itself has several limitations: i) It is largely hypothetical, with most mechanistic links derived from elderly or non-GI cancer contexts; ii) the relative contribution of each axis component to AYA GI cancer risk is unknown; iii) the framework does not account for genetic predisposition (such as Lynch syndrome and familial adenomatous polyposis), which may be overrepresented in young-onset cases; and iv) the model assumes that the observed trends have a biological basis, when they may partly reflect changing environmental exposures that do not involve immunosenescence. It is emphasized that the M-I-H axis should be viewed as a working hypothesis to generate testable predictions, not as an established causal model.

### Conclusion

AYA GI cancers pose a growing clinical challenge that may be driven, at least in part, by premature immunosenescence rather than simple accumulation of mutational events over time. The M-I-H axis offers a potentially useful conceptual framework for exploring how metabolic, inflammatory, hormonal and microbial factors might converge to accelerate immune aging. However, it was emphasized that this model remains largely hypothetical, with numerous proposed associations awaiting direct experimental validation in AYA populations. Throughout the present systematic review, the strength of evidence was annotated to distinguish established findings from speculative extensions.

### Testable hypotheses and proposed study designs

To advance the field beyond speculation, the following specific, testable hypotheses with concrete study designs are proposed.

*Hypothesis 1*. AYA GI cancer patients exhibit accelerated immune aging biomarkers compared with age-matched healthy controls. Proposed study: A cross-sectional biomarker study measuring CD28^-^ CD57^+^ T cells, TREC levels, p16ᴸᴺ_4_ᵃ expression, telomere length, and epigenetic aging indices in AYA patients with GI cancer (n=100), age-matched healthy controls (n=100), and elderly patients with GI cancer (n=100). Primary endpoint: Proportion of AYA patients with biomarker profiles consistent with accelerated immune aging.

*Hypothesis 2*. The M-I-H axis components are intercorrelated in AYA GI cancer patients and collectively predict immunotherapy response. Proposed study: A prospective translational cohort (n=150) of AYA GI cancer patients receiving immunotherapy, with baseline measurement of metabolic markers (leptin, insulin, cholesterol, and lactate), inflammatory markers (IL-6, IL-1β, and SASP factors), hormonal markers (cortisol rhythm, estrogen, and thyroid panel) and microbiome composition. Primary endpoint: correlation of composite M-I-H score with objective response rate.

*Hypothesis 3*. Metabolic modulation (statin + metformin) combined with anti-PD-1 therapy enhances outcomes in AYA patients with MSI-H/dMMR colorectal cancer. Proposed study: A Phase II randomized trial of pembrolizumab ± atorvastatin/metformin in AYA (15-39 years) MSI-H/dMMR colorectal cancer, stratified by baseline leptin levels and CD28^-^ CD57^+^ T-cell frequency. Primary endpoint: progression-free survival. Secondary endpoints: objective response rate, immune-related adverse events and quality of life.

*Hypothesis 4*. Microbiome composition modulates the M-I-H axis and predicts treatment response in AYA GI cancer. Proposed study: A microbiota-stratified AYA cohort receiving standard immunotherapy, with longitudinal stool sampling and metabolomic profiling. Primary endpoint: Association between microbial diversity/metabolite profiles (SCFAs, and secondary bile acids) and treatment response.

Several additional priorities emerge for future research. First, validated plasma biomarkers capable of identifying high-risk AYA individuals before clinical cancer presentation are required. Secondly, clinical trials specifically designed for young-onset populations, rather than post-hoc subgroup analyses, must evaluate whether metabolism-targeted interventions enhance immunotherapy outcomes. Thirdly, longitudinal studies tracking microbiota-hormone-immune interactions from adolescence through early adulthood could identify critical intervention windows.

Ultimately, moving beyond age-based treatment paradigms toward truly personalized oncology for young adults will require mechanistic insights that we are only beginning to glimpse. The M-I-H axis, if validated, could provide a framework for such personalization, but we must be clear-eyed about the gap between hypothesis and evidence. It is hoped that the present systematic review prompts further investigation into this underappreciated clinical problem.

## Figures and Tables

**Table I:** Therapeutic targets in the M-I-H axis for AYA gastrointestinal cancer.

Axis component	Molecular target	Intervention strategy	Clinical status and evidence grade	AYA-specific evidence
Metabolic	HMGCR	Statins (simvastatin, atorvastatin)	Phase II/III trials ongoing; observational data only (evidence moderate)	No AYA-specific trials; hypothesized responsive subgroup in dyslipidemic young patients
Metabolic	Insulin/PI3K	Metformin	Phase II trials in multiple cancers (evidence moderate)	No AYA-specific data; obesity prevalence rising in AYAs supports relevance
Metabolic	Leptin signaling	Leptin receptor antagonists	Preclinical (evidence preclinical only)	No AYA-specific data; testable hypothesis in obese AYA patients
Metabolic	EV biogenesi (nSMase2)	GW4869 (research tool; next-generation inhibitors needed)	Preclinical; GW4869 not viable clinically due to poor pharmacokinetics (evidence preclinical only)	No AYA-specific data; no clinical trials planned
Inflammatory	IL-1α/β	Anakinra, canakinumab	Phase III (CANTOS trial in lung cancer) (evidence robust for lung cancer; untested in GI cancer)	No AYA-specific data; long-term safety in young patients unknown
Inflammatory	IL-6	Tocilizumab	Approved for RA; oncology trials ongoing (evidence moderate in esophageal SCC)	No AYA-specific data; long-term infection risk in young patients a concern
Hormonal	Cortisol/HPA axis	Selective glucocorticoid modulators; stress reduction	Early phase (evidence preclinical/early clinical)	No AYA-specific data; psychosocial stress highly prevalent in AYAs
Hormonal	Estrogen/ER	SERMs (tamoxifen); hormonal modulation	Approved in breast cancer; not tested in GI cancer (evidence preclinical for GI)	No AYA-specific data; fertility considerations critical
Microbial	Gut dysbiosis	Fecal microbiota transplantation; probiotics; dietary intervention	Phase I/II in melanoma (evidence moderate in melanoma)	No AYA-specific data; anatomical relevance to GI cancers supports testing
Immune checkpoint	PD-1/PD-L1	Pembrolizumab, nivolumab	Approved; biomarker-guided (evidence robust)	Limited AYA subgroup data; younger age may correlate with enhanced response

AYA, adolescents and young adults; GI, gastrointestinal; M-I-H, metabolic-inflammatory-hormonal; HMGCR, 3-hydroxy-3-methylglutaryl-CoA reductase; PI3K, phosphoinositide 3-kinase; EV, extracellular vesicle; nSMase2, neutral sphingomyelinase-2; IL-1α/β, interleukin-1α/β; IL-6, interleukin-6; HPA, hypothalamic-pituitary-adrenal; ER, estrogen receptor; SERMs, selective estrogen receptor modulators; PD-1, programmed cell death protein 1; PD-L1, programmed death-ligand 1; SCC, squamous cell carcinoma; RA, rheumatoid arthritis.

## Data Availability

Not applicable.
